# Supplementary material for: An Outbreak of Subclinical Mastitis in a Dairy Herd Caused by a Novel Streptococcus canis Sequence Type (ST55)
Source: Animals (Basel). 2021 Feb 20;11(2):550. doi: 10.3390/ani11020550 (PMC7923261; doi:10.3390/ani11020550)
Supplement: Supplementary file 1 [file animals-11-00550-s001.pdf]

Supplementary material

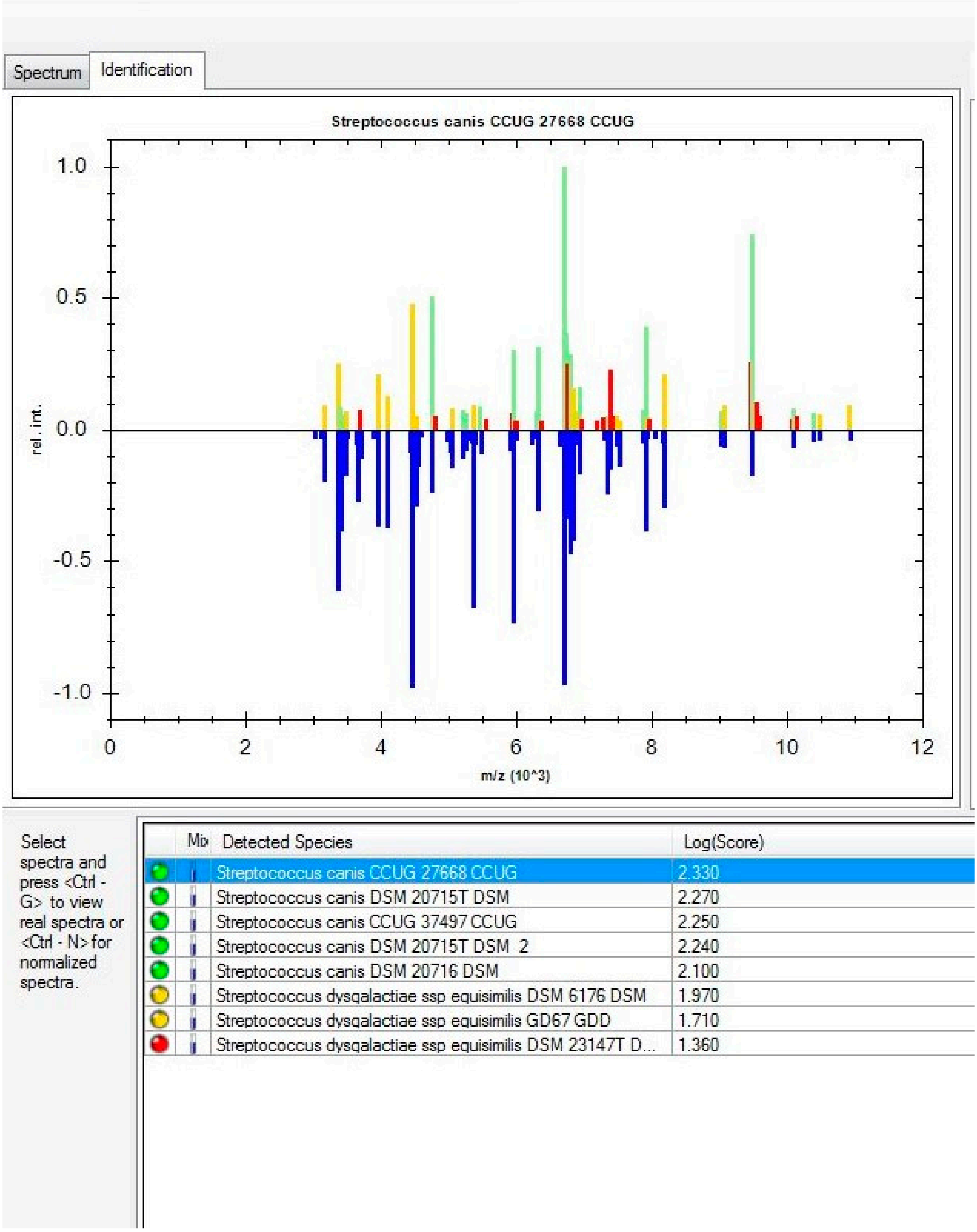

Figure S1. Identification of a streptococcal isolate as *S. canis* using MALDI-TOF mass spectrometry producing log scores above 2.000.
